# Metabolic Fingerprint in Childhood Acute Lymphoblastic Leukemia

**DOI:** 10.3390/diagnostics14070682

**Published:** 2024-03-24

**Authors:** Maria T. Papadopoulou, Paraskevi Panagopoulou, Efstathia Paramera, Alexandros Pechlivanis, Christina Virgiliou, Eugenia Papakonstantinou, Maria Palabougiouki, Maria Ioannidou, Eleni Vasileiou, Athanasios Tragiannidis, Evangelos Papakonstantinou, Georgios Theodoridis, Emmanuel Hatzipantelis, Athanasios Evangeliou

**Affiliations:** 1 4th Pediatric Department, Papageorgiou General Hospital, Aristotle University of Thessaloniki, Papageorgiou General Hospital, Ring Road, Nea Efkarpia, 56403 Thessaloniki, Greece; vivianpa@icloud.com (P.P.); aeevange@auth.gr (A.E.); 2 Woman-Mother-Child Hospital, University Hospitals of Lyon, 69500 Bron, France; 3 NEOLAB S.A., Medical Laboratory, 11527 Athens, Greece; efiparamera@hotmail.com (E.P.);; 4 Department of Chemistry, Aristotle University of Thessaloniki, 54635 Thessaloniki, Greece; al_pechliv@hotmail.com (A.P.);; 5 BIOMIC_Auth, Center for Interdisciplinary Research of the Aristotle University of Thessaloniki (CIRI), Balkan Center, 10th Km Thessaloniki-Thermi Rd, P.O. Box 8318, 57001 Thessaloniki, Greece; 6 Analytical Chemistry Laboratory, Department of Chemical Engineering, Aristotle University of Thessaloniki, 54636 Thessaloniki, Greece; 7 Department of Pediatric Oncology, Ippokratio General Hospital, 54642 Thessaloniki, Greece; 8 Pediatric & Adolescents Hematology-Oncology Unit, 2nd Pediatric Department, AHEPA Hospital, Aristotle University of Thessaloniki, 54636 Thessaloniki, Greece; palabou.m@gmail.com (M.P.); ioannidou_m@hotmail.com (M.I.); atragian@auth.gr (A.T.); hatzip@auth.gr (E.H.); 9 St Luke’s Hospital S.A., 55236 Pannorama, Greece

**Keywords:** metabolomics, fatty acids, amino acids, organic acids, therapeutic metabolic pathways

## Abstract

Introduction: Acute lymphoblastic leukemia (ALL) is the most prevalent childhood malignancy. Despite high cure rates, several questions remain regarding predisposition, response to treatment, and prognosis of the disease. The role of intermediary metabolism in the individualized mechanistic pathways of the disease is unclear. We have hypothesized that children with any (sub)type of ALL have a distinct metabolomic fingerprint at diagnosis when compared: (i) to a control group; (ii) to children with a different (sub)type of ALL; (iii) to the end of the induction treatment. Materials and Methods: In this prospective case–control study (NCT03035344), plasma and urinary metabolites were analyzed in 34 children with ALL before the beginning (D0) and at the end of the induction treatment (D33). Their metabolic fingerprint was defined by targeted analysis of 106 metabolites and compared to that of an equal number of matched controls. Multivariate and univariate statistical analyses were performed using SIMCAP and scripts under the R programming language. Results: Metabolomic analysis showed distinct changes in patients with ALL compared to controls on both D0 and D33. The metabolomic fingerprint within the patient group differed significantly between common B-ALL and pre-B ALL and between D0 and D33, reflecting the effect of treatment. We have further identified the major components of this metabolic dysregulation, indicating shifts in fatty acid synthesis, transfer and oxidation, in amino acid and glycerophospholipid metabolism, and in the glutaminolysis/TCA cycle. Conclusions: The disease type and time point-specific metabolic alterations observed in pediatric ALL are of particular interest as they may offer potential for the discovery of new prognostic biomarkers and therapeutic targets.

## 1. Introduction

Acute lymphoblastic leukemia (ALL) is the most common malignant disease (25% of all cases) in patients aged < 15 years [1,2]. While advances in the understanding of the disease and the implementation of appropriate treatment regimens have improved survival rates to 89.4% in developed countries [2,3], relapsed ALL remains a significant cause of mortality, particularly in adolescents [3,4,5]. Refractory and relapsed leukemia are mainly associated with the resistance that leukemic cells develop, in some cases, to the most commonly used chemotherapeutic agents [6,7]. As a result, there has been a long-standing interest in finding biomarkers that can predict the likelihood or occurrence of relapse in a timely manner, as well as identifying novel targets for innovative therapies [7,8]. 

Until recently, cancer research has focused primarily on the genome and proteome, providing important knowledge about the biology of cancer cells, but leaving gaps regarding the various final metabolic expressions of genes and proteins [9]. These gaps have been bridged in recent years by the ever-growing interest in metabolomics, which corresponds to the ultimate expression of genes and proteins in cellular metabolism, reflects all changes in their phenotype and function, and represents the final functional and biochemical aspect of any biological system in health and disease [9,10,11,12,13]. 

The first biochemical change found in cancer cells was the increased rate of aerobic glycolysis, known as the Warburg effect, which was described by biochemist Otto Warburg in 1924 [14]. Following Warburg’s initial experiments, a plethora of studies have shown that cancer cells’ mitochondria are smaller and have a reduced surface area as a substrate of OXPHOS, while some tumor cells also have significantly fewer mitochondria compared to healthy ones [15,16,17]. Since then, there has been continuous research on the topic and on the potential application of metabolomics in clinical oncology and hematological malignant diseases [18,19,20,21].

In vivo data supporting metabolic changes of the cells in pediatric ALL are limited. The aim of this study was to use targeted metabolomics analysis to investigate whether there are alterations in children with ALL compared to controls. We further hypothesized that there are distinct changes in the metabolism of patients according to the ALL subtype and the time point of the disease, at diagnosis and before the start of the chemotherapy (reflecting the effect of cancer cell proliferation and metabolism) and at the end of induction therapy (reflecting the effect of chemotherapy and the destruction of cancer cells). 

## 2. Methods

### 2.1. Study Protocol 

This prospective, case–control study (Clinical trials study identifier: NCT03035344) was conducted as part of a PhD thesis and approved by the Bioethics Committee of the Aristotle University (216/23 March 2016). Inclusion criteria for patients were as follows: newly diagnosed acute lymphoblastic leukemia; age 1–16 years; absence of any other underlying chronic disease. 

### 2.2. Enrollment 

Enrollment took place over three years at three sites in Thessaloniki, Greece. The diagnosis, risk group stratification, and subsequent treatment regimen for children with ALL were based on the ALL IC-BFM 2009 protocol [22]. 

The control group was prospectively enrolled during the same period and included children of similar age (±6 months) and the same sex as the patients, without malignant or inflammatory diseases. 

The study was conducted in accordance with the Declaration of Helsinki, and approved by the Ethics Committee of the Medical School of Aristotle University of Thessaloniki (216/26 March 2016). Written informed consent was obtained from the parents or guardians of all subjects involved in the study. 

### 2.3. Sample Collection

Blood and urine samples were collected from patients and controls after a 12-h overnight fast. A heparinized syringe was used to collect blood samples, which were then transferred to empty vials. They were left at room temperature for one hour, and then the supernatant plasma was collected after centrifugation at 4000 rpm (4 °C) for 10 min. Plasma was placed in special plastic storage vials with plastic caps and stored in a refrigerator at −20 °C. Urine samples (10 mL of fresh urine in plastic containers) were also stored at −20 °C.

Patients were sampled at two time points, before the start of induction chemotherapy (D0) and at the end of induction chemotherapy (D33).

### 2.4. Sample Analysis 

The determination of metabolites in patients and controls was performed at NEOLAB, S.A according to the internal and external quality control procedures of the laboratory. The metabolic profile obtained included the following analyses: quantification of plasma and urine amino acids, plasma total fatty acids, plasma carnitine esters and urine organic acids. Gas chromatography–mass spectrometry (GC–MS) was used to identify and quantify urine organic acids and plasma fatty acids, using an Agilent 7890A-5875C GC-MS [23,24,25]. Plasma acylcarnitines, plasma and urine amino acids were analyzed by liquid chromatography–tandem mass spectrometry (LC–MS/MS) [26,27]. Quantification of all metabolites was based on the elution time and peak height of each metabolite.

### 2.5. Data Analysis

We collected GC–MS and LC–MS/MS-quantified metabolomics data for 22 plasma carnitine esters, 20 plasma and urine amino acids, 20 plasma fatty acids and 44 urine organic acids. 

The biostatistical-bioinformatic analysis was performed in collaboration with the Department of Chemistry and the Center for Interdisciplinary Research and Innovation (*K.E.D.E.K*) of Aristotle University of Thessaloniki. Multivariate statistical analysis included principal component analysis (PCA) and was performed using SIMCA 13.0.3 software (UMETRICS AB Sweden). The data were further processed by applying partial and orthogonal-partial least squares discriminant analysis (PLS-DA, OPLS-DA). The components with important contribution to the metabolomic profiling models were identified by the “Loadings Plot” and “S-Plot” method using the absolute cut-off values of p and p (corr) as limits. Of these, only parameters with variable importance in projection (VIP) value > 1 and *p*-value < 0.05 were considered important. 

Model quality was assessed by a test for good adaptation to the variables X (R2X) and Y (R2Y) and the predictability (Q2YCV) by permutation and by analysis of variance testing of cross-validated predictive residuals (CV-ANOVA). Univariate statistical analysis was performed with a two-tailed *t*-test for unequal variances to highlight any differences in the metabolites between the groups studied (the limit of statistical significance was defined as *p*-value < 0.05).

Visualization of changes and variances of potential biomarkers in each group was achieved with boxplots generated using the R programming language. The KEGG database (URL https://www.genome.jp/kegg/compound accessed on 25 October 2023) was used to define metabolite networks and pathways. 

## 3. Results

### 3.1. Participants

Thirty-seven patients aged 1–16 years and an equal number of controls of comparable age (±6 months) and sex were initially included in the study. Three patients were excluded from the analysis because the final diagnosis was acute myeloid leukemia (AML). The median age of the 34 remaining patients was five years old (range: 1–16 years, IQR: 7). Eighty-two per cent (82.4%) of the participants were male. Samples from both pre- and post-induction chemotherapy were available in 22 patients (64.7%). All patients followed the same induction protocol treatment, regardless of their risk group. The baseline characteristics of the patients are shown in Table 1.

### 3.2. Metabolomic Multivariate Analysis

**Figure 1:**
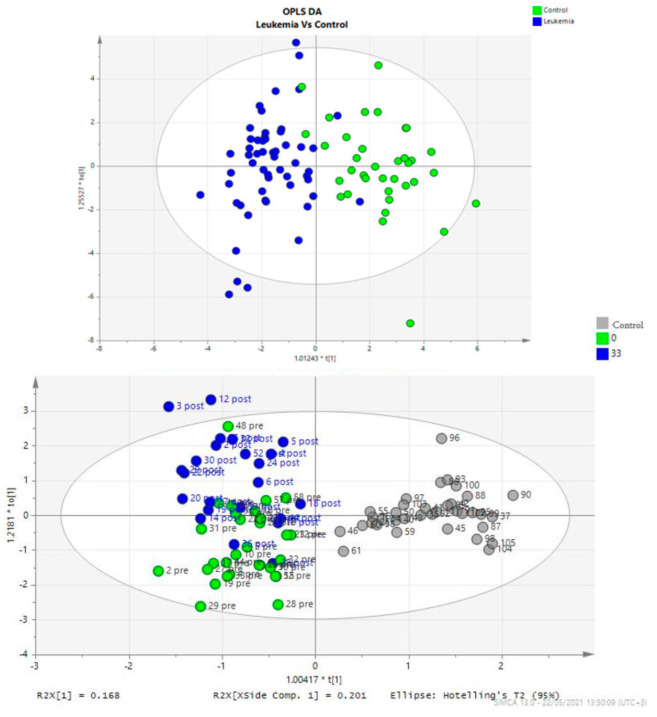
Visualization of the distinct metabolic fingerprint after combined multifactorial statistical analysis of all metabolites of ALL patients and controls. (**Upper**): Urine metabolites OPLS−DA plots separation between patients (left side of the t1 axis, blue dots) and controls (right side of the t1 axis, green dots). (**Lower**): Plasma metabolites OPLS−DA plots separation between patients (left side of the t1 axis, colored dots) and controls (right side of the t1 axis, grey dots). Dots corresponding to the metabolic fingerprint of patients at diagnosis (D0, green dots) are localized in the lower left quartile of the plot while the metabolic fingerprint data at the end of the induction treatment (D33, blue dots) are concentrated in the upper left quartile.

**Figure 2:**
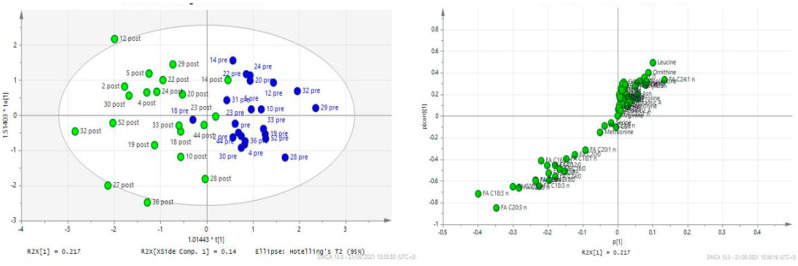
(**Left**): Visualization of the distinct metabolic fingerprint after combined multifactorial statistical analysis of all plasma metabolites of patients with ALL at diagnosis (blue dots, D0) and at the end of induction (D33, green dots). (**Right**): Loadings plot analysis showing the plasma metabolites that contributed most to the difference in the metabolomic fingerprint of patients at diagnosis compared to D33. The major contributors are those with the greatest distance from the (0, 0) point and in particular increases in carnitine, specific aminoacids as leucine and ornithine and lower levels of polyunsaturated fatty acids.

Figure 1 provides a visualization of the plasma metabolic fingerprint of patients compared to controls, with a clear distinction of the plasma metabolic fingerprint not only between patients and controls, but also between patients at diagnosis and at the end of induction treatment. The latter is further illustrated in Figure 2, along with an additional analysis showing the major contributors (plasma metabolites) to this divergence.

The above analysis also tested for the possible contributions of patient age, gender, BMI, risk group at diagnosis and initial remission (on D33). There was no change in the results after adjustment for the above factors (*p* > 0.05). 

**Figure 3:**
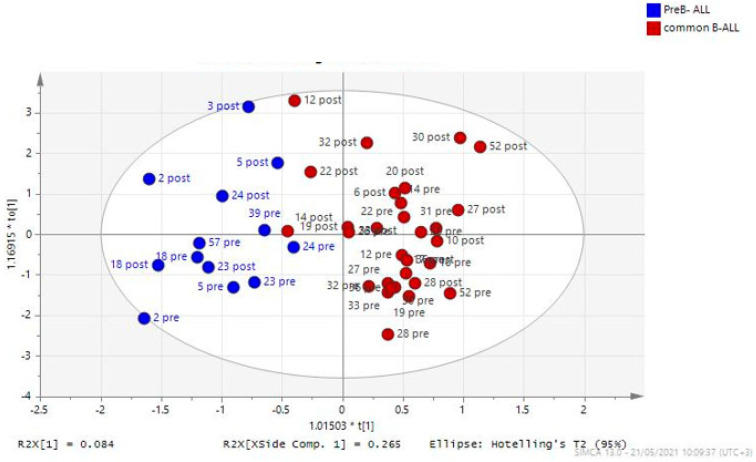
Visualization of the distinct metabolic fingerprint with OPLS-DA combined multifactorial analysis of plasma metabolites between patients with common B-ALL (red dots) and pre-B-ALL (blue dots). Further separation of the metabolomic profile according to the time point of their treatment is represented by the accumulation of the fingerprints at diagnosis (“pre”) at the two upper quartiles of the figure, while the dots representing the metabolic fingerprint of the patients at the end of induction (“post”) are located in the lower quartiles of the plot.

The multivariate analysis also allowed the designation of a distinct metabolic fingerprint according to the subtype of B-ALL, as illustrated in Figure 3. The metabolites that mainly contributed to the difference between common B-ALL and pre-B ALL were higher levels of decanoylcarnitine, docosahexaenoic acid (DHA, omega-3 fatty acid), threonine, valine, lysine and isoleucine in patients with common B-ALL compared to pre-B ALL patients. In contrast, patients with pre-B ALL had higher levels of myristic acid and methionine.

### 3.3. VIP and Univariate Analysis

The relative contribution of each plasma metabolite to the metabolic fingerprint of the multifactorial OPLS-DA models was further tested by VIP analysis. Plasma metabolites with VIP > 1 are presented in Table 2. The metabolites with the higher contribution (VIP > 2) to the alteration of the plasma metabolic fingerprint along with their log2(FC) patients/controls were as follows: free and acetyl-carnitine (C0 (0.92); C2:0 (1.04)), as well as myristic acid (saturated FA) (1.48), palmitelaidic acid (mono-unsaturated FA) (1.6), and dihomo-γ-linolenic acid (DGLA, poly-unsaturated omega-6 FA) (1.22). 

**Figure 4:**
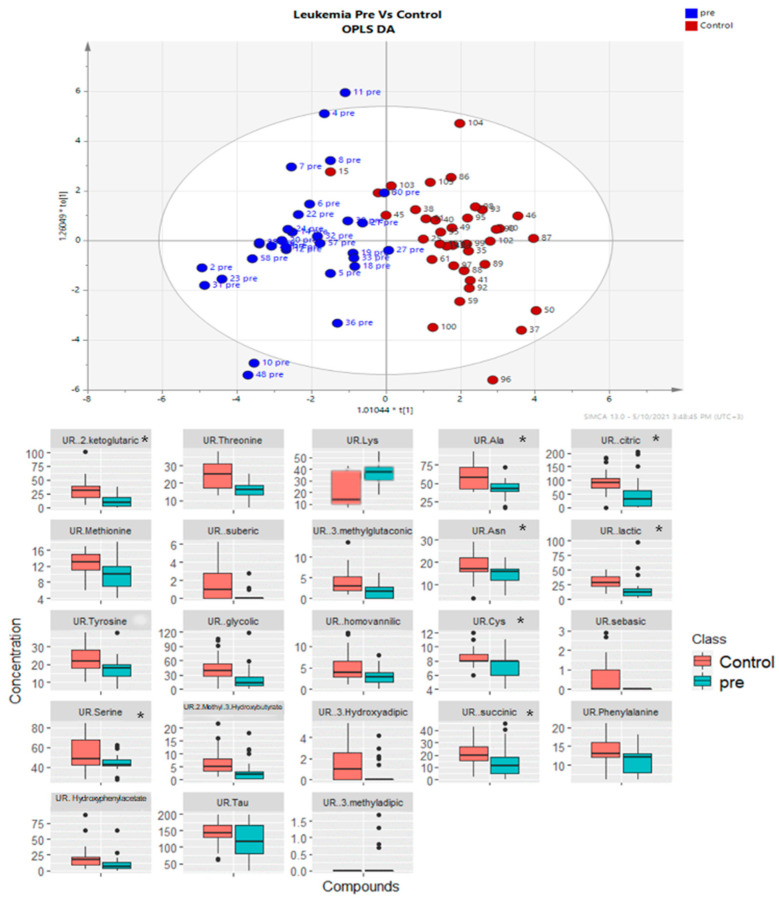
(**Upper**) Visualization of the distinct metabolic fingerprint with OPLS-DA combined multifactorial analysis of urine metabolites of patients with ALL (blue dots) at diagnosis and before the start of the induction compared to controls (red dots). (**Lower**) Boxplots showing the urine compounds with significant differences (*t*-test, sp < 0.05) between ALL patients at diagnosis and compared to controls. The asterisk * indicates compounds involved in or related to glutamine metabolic pathways (e.g., glutaminolysis), suggesting an important role of this pathway in the altered metabolism of cancer cells. Lys = lysine, Ala = alanine, Asn = asparagine, Cys = cysteine, Tau = taurine.

Comparisons of metabolite levels between ALL patients at diagnosis and controls are presented in Figure 4, along with the OPLS-DA multivariate model of the urine metabolite analysis. The effect size of the difference (log2(FC) patients at diagnosis/controls) showed that patients had significantly (*p* < 0.05) lower concentrations for the following amino acids: threonine (−0.55), alanine (−0.46), methionine (−0.38), asparagine (−0.35), tyrosine (−0.35), cysteine (−0.2), serine (−0.2), phenylanine (−0.24), taurine (−0.25), and higher levels of lysine (0.61). Patients had significantly lower concentrations for the following organic acids: 2-ketoglutaric acid (−1.37), citric acid (−0.96), suberic acid (−2.98), 3-methylglutaconic acid (−1.06), lactic acid (−0.78), glycolic acid (−0.93), homovanillic acid (−0.77), 2-methyl-3-hydroxybutyric acid (−0.99), 3- hydroxy-adipic acid (−1.73), succinic acid (−0.57), 4-hydroxyphenylacetic acid (−0.87), and 3-methyladipic acid (−0.76). Eight out of a total of 23 of the above metabolites are involved in the metabolic pathway of glutamine and its derivatives, as detailed in Figure 4.

## 4. Discussion

In this study, we used a targeted metabolomic analysis that has permitted the identification of a distinct metabolic fingerprint of children with ALL at diagnosis and after the initiation of chemotherapy; both time points were also compared to age- and sex- matched controls. We defined the metabolites that mainly contribute to this difference, providing insights into the metabolic rewiring of leukemic cells based on an in vivo metabolomic study. The results of the sub(group) analysis supported the concept that different ALL types result in distinct changes in urine and plasma metabolites. 

We hereby provide an overview of the literature and compare the existing evidence to the main metabolomic changes found in our study population, along with the therapeutic implications of these findings if further confirmed.

### 4.1. Metabolomics in Hematological Malignancies 

The use of metabolomics in hematological malignant diseases has shown very promising results and applications to novel therapies [28]. A study by MacIntyre and colleagues highlighted for the first time in 2010 the potential role of the metabolome as a prognostic tool for identifying biomarkers related to staging and outcome of patients with chronic lymphocytic leukemia [29]. Similarly, Puchades-Carrasco and colleagues recorded significant differences in the amino acids and lipids of patients at initial diagnosis of multiple myeloma compared to a control group, as well as a shift in the metabolic profile of patients after disease remission to a pattern similar to that of controls [30]. Wang and colleagues analyzed in a prospective study the characteristics of the intermediate metabolism in patients with acute myeloid leukemia and observed significant differences in serum amino acids, choline, lactate and lipids, not only between patients and controls, but also between patients with favorable and intermediate risk factors [31].

### 4.2. Metabolomics in ALL

The available evidence for metabolomic alterations in pediatric ALL is, to date, limited; however, there are a few pediatric/adult studies showing promising results for biomarker identification [32,33] regarding disease phenotyping, progression and prognosis [34,35,36,37], as well as treatment response and the related side effects [38,39,40,41]. In addition, several studies have shown that metabolic changes during inflammation and fever tend to be specific for the etiopathogenic agent, raising hopes for potential metabolic biomarkers that could differentiate leukemia from leukemia-mimicking disorders in the pediatric population [42,43,44,45]. 

### 4.3. Fatty Acid Alterations

Table 2 demonstrates that a substantial part of the difference in the metabolic profile of patients with ALL compared to controls was due to the increase in several saturated and unsaturated fatty acids. Specifically, the major contributors were myristic and palmitic acids and their derivatives. They are among the most common FA found in the human body and are mainly involved in lipid biosynthesis and energy production through mitochondrial b-oxidation. In vitro studies of cancer cells have confirmed at the cellular level important alterations in their lipogenesis [46]. These alterations may be associated with the enzyme complex of fatty acid synthase (FASN). In particular, the increased metabolism of glucose leads to an excess of acetyl-CoA. Rapidly proliferating cancer cells then use the excess acetyl-CoA, via FASN and a second important enzyme, acetyl-CoA carboxylase (ACC), for de novo synthesis of lipids, which are the building blocks of the cell membranes of new cancer cells. Most healthy cells preferentially use the lipids provided by the diet for the normal production of new structural lipids, whereas cancer cells favor their de novo endogenous synthesis through the overexpression of FASN [47]. The above mechanism, which seems to be closely related to cancer cell survival, has been confirmed in cancer cells from various tumors (mainly epithelial) of the genitourinary and digestive systems [48].

There are also in vivo metabolomic data supporting the key role of lipid metabolism rewiring in ALL. Bai et al. [33] identified 30 metabolites that could potentially serve as disease biomarkers, most of which were involved in the pathway of glycerophospholipids, but also in the a-linolenic acid metabolism. Many of the FA involved in the latter were also found to be elevated in ALL patients in our study. A more recent study in a pediatric ALL population further supported the theory that the increase in fatty acids is attenuated at the end of the induction treatment [38]. Our analysis also showed that the lipid metabolism remains altered compared to the control group at the end of the induction treatment.

### 4.4. Carnitine and Esters

Plasma carnitine and short-chain acetylcarnitines were increased in patients compared to controls and remained higher at the end of the induction chemotherapy, although the difference was attenuated. Given the need for excessive energy production from fatty acids, this result is not surprising, since carnitine and its acetylcarnitines have a central role in the transport of fatty acids in the mitochondrion and in β-oxidation. Fatty acyl-CoA production is mediated by the following four main enzymes: fatty acyl-CoA synthetase (FACS), carnitine palmitoyl transferase 1 (CPT1), carnitine acylcarnitine translocase (CACT), and carnitine palmitoyl transferase 2 (CPT2) [49]. Inhibitors of CPT1 have already shown in vitro efficacy in reducing proliferation of chronic lymphocytic leukemia cells [50]. Based on our data, this pathway warrants additional investigation in ALL too. 

### 4.5. Organic and Amino Acids and Alterations 

Taurine, serine and proline were the plasma amino acids that showed the most important alterations between patients and controls according to multivariate analysis, while lower levels of several other amino acids were also identified. Yang et al. reported significant differences in adult patients with B-ALL before the start of treatment compared to healthy controls in various pathways (glycolysis, gluconeogenesis, metabolism of amino acids, fatty acids and choline phospholipids metabolism) and proposed the combination of choline, tyrosine and unsaturated fatty acids as a diagnostic indicator that can distinguish patients with B-ALL from patients without B-ALL [51]. Although the number of patients in the aforementioned study was too small to draw firm conclusions, some of these changes were also observed in our sample. In particular, although tyrosine was not among the amino acids with the highest contribution in plasma analysis, urine tyrosine levels were significantly lower compared to controls. Moreover, serine (a precursor molecule of choline) was among the amino acids with the greatest reduction in patients compared to controls in both plasma and urine samples. This difference remained statistically significant even after the end of induction, despite a relative increase in the plasma levels of serine. Serine and its various derivatives are involved in numerous cell functions and pathways in the human body, including formation of cell membranes and cellular apoptosis [52,53]. Along with choline, they are also involved in the metabolic pathway of glycerophospholipids, the major component of cellular membranes.

Glutaminolysis is upregulated in many types of cancer, and glutamine provides these rapidly proliferating cells with a crucial source of nitrogen for amino acid synthesis via glutamate production and transamination [54]. Glutamine metabolism also appears to be a key pathway in the metabolic changes in ALL patients, according to the amino acid analysis in our study, which was further supported by the urinary organic acid analysis. Specifically, 2-ketoglutaric, citric and succinic acids, which were significantly decreased in the patient group, are metabolites of the tricarboxylic acid (TCA) cycle. Glutaminolysis produces 2-ketoglutaric acid that replenishes the TCA cycle, and not only provides intermediates for other biosynthetic pathways, but also supports cell growth and energy production [55,56]. The ability of cancer cells to coordinate enhanced glycolysis and mitochondrial TCA to meet their increased energy needs is under study [56,57], and our data further support its relevance in pediatric ALL with therapeutic prospects.

### 4.6. Therapeutic Implications of Metabolomic Pathways in Leukemia

There has been great interest in investigating the metabolic vulnerabilities of leukemic cells, with particular emphasis on their potential application at the therapeutic level [58,59]. The majority of studies have focused on leukemic cells of the myeloid lineage, possibly because of their significant resistance to treatment [28]. To date, we have data related to lipid metabolism and other metabolic pathways that may pave the way for possibilities of targeted metabolic treatments in these patients [60,61]. 

Wang and colleagues demonstrated metabolic alterations in multiple pathways and defined a characteristic “signature” of glucose metabolism in adult patients with acute myeloid leukemia by highlighting six metabolic biomarkers with predictive value [31]. Subsequently, they showed in an in vitro stem cell analysis that this alteration was prognostic for sensitivity and cytotoxicity to the antileukemic agent cytarabine (Ara–C) [62]. Pei and colleagues have pointed out a discrepancy in glutathione metabolism in acute myeloid leukemia cells and then achieved cell death by interfering with the glutathione pathway [63].

Regarding lipid metabolism, Stuani and colleagues studied acute myeloid leukemia cells with a mutation in the enzyme isocitrate dehydrogenase (IDH1), which is common in these resistant cancer cells, and demonstrated the association of the mutation with the impaired lipid anabolism in these cells [64]. The increased levels of many saturated and unsaturated fatty acids in ALL patients in our controlled study suggest a similar enhancement of lipid synthesis in the leukemic cells, resulting in their accumulation and increased circulation in the plasma. According to a recent experimental study, adipocytes appear to be involved in this mechanism, highlighting their importance as a potential therapeutic target [65]. Furthermore, siramesine, a cationic amphiphilic drug (CAD) has been shown to be effective in accelerating cell death in ALL and AML cell lines, by altering the lipid metabolism of cancer cells, specifically by increasing lysoglycerophospholipids [66]. Our results provide further in vivo evidence of enhanced lipid metabolism in ALL, and the discovery/repositioning of drugs that interfere with/inhibit lipid metabolism should be further explored. 

The combination of metabolomics and other omics techniques may further advance the therapeutic perspectives of metabolic rewiring. For example, we have already mentioned the therapeutic potential of the glutamine pathway, as it plays multiple roles in cancer cell survival as an essential metabolite for mitochondrial energy production, reactive oxygen species scavenging, cell signaling activation, and synthesis of non-essential amino acids, purines, pyrimidines, and fatty acids [67,68]. Sbirkov et al. used a combined transcriptomic and metabolomic approach to demonstrate dysregulation in several metabolic pathways in B-ALL cells and then showed in vitro efficacy of glutaminolysis rewiring in both sensitive and prednisolone-resistant B-ALL cells [69]. The authors used clinical metadata from relapsed patients to investigate the clinical relevance of their in-vitro observation, with promising results. We provided further in vivo data supporting the prominent role of glutamynolysis in the metabolic dysregulation in B-ALL patients compared to controls, emphasizing the therapeutic potential of rewiring this pathway. 

The main limitation of our study is the small sample size, which was sufficient to answer the main research question but did not allow for robust subgroup analyses of clinical relevance, such as potential further metabolic differences between patients with B-ALL and T-ALL or between patients with good and poor response to induction treatment. However, the sample in our study is comparable or larger than in several studies in the field, and has the additional advantage of a controlled design. Our sample was prospectively collected from an unselected population of patients diagnosed with ALL in Thessaloniki, Greece. No patient/parent refused to participate in the study. Nevertheless, patients who started treatment before the presentation of the study could not be included, resulting in a long recruitment period and probably some imbalances in our sample, such as the large percentage of male participants. We have addressed this point by having a matched control group and by confirming no effect of the baseline characteristics within the patient group in the multivariate models. A solely metabolomics approach was used for this study but a combined genomic–transcriptomic–metabolomic analysis would be the optimal approach for future projects.

## 5. Conclusions

We present the results of a metabolomic analysis in pediatric patients with ALL, confirming alterations in their intermediate metabolism, resulting in a unique pattern that differs between patients and controls, and also between patients with pre-B and common B-ALL. This metabolic fingerprint is further altered, but remains distinct compared to controls, at the end of the induction treatment, a critical time point for the evolution of the disease at which the initial response to treatment is evaluated. Our analysis successfully identified metabolites, mainly involved in fatty acid synthesis, transfer and oxidation, in amino acid and glycerophospholipid metabolism and in the glutaminolysis/TCA cycle. The above-mentioned pathways merit further in vitro and in vivo investigation and multi-omics approaches, which may lead to novel biomarkers and therapeutic targets in childhood ALL. 

## Figures and Tables

**Table 1:** Baseline demographic and leukemia related information of patients included in the analysis.

Patients (N = 34)				
Age ^a^	Median in years (IQR)			
	5 (7)			
Sex (N, %)	*Female*		*Male*	
	6 (17.6%)		28 (82.4%)	
BMI ^a^	*Median in kg/m^2^ (IQR)*			
	16 (5.4)			
Type of ALL (N, %)	*Common B-ALL*	*Pre-B ALL*		*T-ALL*
	20 (58.8%)	8 (23.5%)		6 (17.6%)
Risk group ^a^ (N, %)	*Low*	*Intermediate*		*High*
	14 (41.2%)	16 (47.1%)		4 (11.8%)
Complete initial remission ^b^ (N, %)	*Yes*		*No*	
	27 (79.4%)		7 (20.6%)	

N = number of participants; ALL: acute lymphoblastic leukemia; BMI: body mass index; IQR: interquartile range; ^a^ as defined on diagnosis; ^b^ as defined by the absence of minimal residual disease on ~day 33 of the induction treatment.

**Table 2:** Results of the variable importance in projection (VIP) analysis of plasma metabolites with VIP score > 1, representing the metabolites with the most important relative contribution to the OPLS-DA metabolomic separation between patients and controls along with the estimate of the effect size of each metabolite as shown by the log(2)fold change (*p* < 0.05).

Metabolite (Plasma)	VIP	Log2fc (Patients/Controls)
**Carnitines**		
C0 (Carnitine)	2.62	0.98
C2:0 (Acetylcarnitine)	2.02	1.04
C16:0 (Palmitoylcarnitine)	1.56	1.26
C3:0 (Propionylcarnitine)	1.33	1.01
**Fatty acids**		
***Saturated***		
Myristic acid	2.32	1.43
Lauric acid	1.5	0.8
Palmitic acid	1.22	0.48
***Mono-unsaturated***		
Palmitelaidic acid	2.1	1.6
Nervonic acid	1.62	−0.7
Myristoleic acid	1.6	1.76
***Polyunsaturated***		
Dihom**o-**γ-linolenic acid (DGLA, omega-6)	2. 3	1.22
Docosahexaenoic acid (DHA, omega-3)	1.71	−0.9
Gamma-linolenic acid (GLA- omega-6)	1.54	2.25
Alpha-linolenic acid (ALA, omega-3)	1.33	0.63
Eicosapentaenoic acid (EPA, omega-3)	1.21	0.91
**Aminoacids**		
Taurine	1.23	−0.36
Serine	1.18	−0.34
Proline	1.05	−0.39

VIP: Variable importance in projection analysis; log2fc: log(2)fold changes showing the size of increase (positive values) or decrease (negative values) of each plasma metabolite between patients and controls.

## Data Availability

Data supporting reported results are available from the corresponding author on request.

## Abbreviations

ALL	Acute lymphoblastic leukemia
AML	Acute myeloid leukemia
BMI	Docosahexaenoic acid
C0	Carnitine
C2:0	Acetylcarnitine
C16:0	Palmitoylcarnitine
C3:0	Propionylcarnitine
CACT	Carnitine acylcarnitine translocase
CAD	Cationic amphiphilic drug
CPT1	Carnitine palmitoyl transferase 1
CPT2	Carnitine palmitoyl transferase 2
DHA	Docosahexaenoic acid
DGLA	Dihomo-γ-linolenic acid
FA	Fatty acids
FACS	Acyl-CoA synthetase
GC-MS	Gas chromatography–mass spectrometry
HCl	Hydrochloric acid
LC-MS/MS	Liquid chromatography–tandem mass spectrometry
Log2(FC)	Log2Fold change
OXPHOS	Oxidative phosphorylation
PLS-DA, OPLS-DA	Partial and orthogonal-partial least squares discriminant analysis
PCA	Principal component analysis
TCA	Tricarboxylic acid
VIP	Variable importance in projection
